# Characteristics and Popularity of Videos of Abusive Head Trauma Prevention: Systematic Appraisal

**DOI:** 10.2196/60530

**Published:** 2024-12-10

**Authors:** Luc Goethals, Victoria Prokofieva Nelson, Fabien Fenouillet, Karine Chevreul, Manon Bergerat, Christine Lebreton, Yacine Refes, Flora Blangis, Martin Chalumeau, Enora Le Roux

**Affiliations:** 1 Obstetrical, Perinatal and Pediatric Epidemiology Research team (EPOPé), Centre of Research in Epidemiology and Statistics (CRESS) Université Paris Cité Inserm Paris France; 2 Epidémiologie clinique-évaluation économique appliqué aux populations vulnérables (ECEVE) Université Paris Cité Inserm Paris France; 3 Learning, Physical Activity and Health Interdisciplinary Laboratory in Neuroscience, Physiology and Psychology Université Paris Nanterre Nanterre France; 4 Kastafiore Paris France; 5 Child protection unit, Department of General Pediatrics Necker-Enfants malades hospital, Assistance Publique des Hôpitaux de Paris (AP-HP) Université Paris Cité Paris France; 6 Synakène Ivry-sur-Seine France; 7 Unit of Clinical Epidemiology (CIC-EC 1426) Hôpital Universitaire Robert-Debré, Assistance Publique des Hôpitaux de Paris (AP-HP) Inserm Paris France

**Keywords:** abusive head trauma, child physical abuse, shaken baby syndrome, SBS, primary prevention, web-based videos, digital tools, head trauma, prevention, video, internet, infant, mortality, morbidity, parent, caregivers, communication

## Abstract

**Background:**

Numerous strategies for preventing abusive head trauma (AHT) have been proposed, but controlled studies failed to demonstrate their effectiveness. Digital tools may improve the effectiveness of AHT prevention strategies by reaching a large proportion of the adult population.

**Objective:**

This study aimed to describe the characteristics of videos of AHT prevention published on the internet, including their quality content, and to study their association with popularity.

**Methods:**

From a systematic appraisal performed in June 2023, we identified videos addressing the primary prevention of AHT in children younger than 2 years that were published in English or French on the internet by public organizations or mainstream associations. We analyzed the characteristics of the videos; their quality with the Global Quality Scale (GQS); and their association with an index of popularity, the Video Power Index, using multivariable quasi-Poisson modeling.

**Results:**

We included 53 (6.6%) of the 804 videos identified. Videos were mainly published by public organizations (43/53, 81%). The median time spent on the web was 6 (IQR 3-9) years, the median length was 202 (IQR 94-333) seconds, and the median GQS score was 4 (IQR 3-4). Infants were often depicted (42/53, 79%), including while crying (35/53, 66%) and being shaken (21/53, 40%). The characterization of shaking as an abuse and its legal consequences were cited in 47% (25/53) and 4% (2/53) of videos, respectively. The main prevention strategies in the videos were to raise awareness of the noxious outcome of shaking (49/53, 93%) and convince viewers of the effectiveness of coping strategies for infants’ cries (45/53, 85%). The Video Power Index was positively correlated with the GQS (*r*=0.38; *P*=.007) and was independently associated with depicting an infant being shaken (*P*=.03; β=1.74, 95% CI 1.06-2.85) and the use of text or headers (*P*=.04; β=2.15, 95% CI 1.08-4.26).

**Conclusions:**

AHT prevention videos had high quality but did not frequently deal with parental risk factors. The characteristics identified as being associated with the popularity of AHT prevention videos could help improve the impact of future prevention programs by enhancing their popularity.

## Introduction

Abusive head trauma (AHT), the most severe form of child physical abuse, is defined as “an injury to the skull or intracranial contents of an infant or young child due to inflicted blunt impact and/or violent shaking” [1]. AHT most often occurs in infants younger than 2 years, at a mean age of 2 to 4 months [2-5], and its incidence has been estimated at 14 to 34 per 100,000 infants per year in countries with developed economies (CDE) [6-9]. Perpetrators of AHT are most often adult men acting alone [10]. Persistent crying is reported as the primary trigger for most AHT perpetration [11]. AHT primary prevention is pivotal because its mortality rate ranges from 7% to 26% [12-14], and nonlethal forms are associated with severe long-term morbidity, notably neurodevelopmental impairment [15].

Numerous AHT prevention strategies have been proposed, targeting the public, parents, or caregivers of children through mass communication campaigns (brochures, television, and radio) or specific programs that mainly rely on educating about the management of infants’ cries and the dangers of shaking a child [16,17]. These prevention programs also emphasize emotional regulation, particularly through the promotion of coping strategies when dealing with stress or frustration in front of infants’ persistent crying [16]. The effectiveness of these strategies has been evaluated in several controlled trials, and although some individual studies have reported reductions in AHT incidence [18-20], meta-analyses and the US Preventive Services Task Force have concluded no significant summed preventive effects [16,21-24]. This discrepancy may be due to differences in the study design used to measure the impact of the intervention and the level of proof used to define effectiveness [24]. More generally, despite all these efforts, the incidence of AHT is not declining in CDE, particularly in the wake of the COVID-19 pandemic and its prevention and mitigation measures [25,26]. Consequently, there is a pressing need for innovative approaches that can reach a wider population and lead to effective behavioral change among the parents and caregivers of tens of millions of newborns each year [27].

Public organizations increasingly diffuse prevention videos on wide-audience digital platforms to support and educate new parents [28]. This approach can reach a large part of the population and become an effective vector for health promotion and prevention [29]. Thus, internet videos could participate in the effective prevention of AHT [30,31]. Young adults are major consumers of internet videos [32] and frequently use the internet to search for health information [33,34]. Consequently, knowing the characteristics of AHT prevention videos that are associated with a wide reach (ie, popularity) will help build more impactful public health interventions. Although measuring the direct impact of such videos on the incidence of AHT is complex and requires large-scale studies [35,36], understanding the factors that contribute to their popularity can serve as an intermediate step toward developing innovative prevention programs. The effectiveness of these programs could ultimately be evaluated by the reduction in AHT incidence. We aimed to describe the characteristics of videos of AHT prevention published on the internet, including their quality content, and to study their association with popularity.

## Methods

### Ethical Considerations

This study was determined to be exempt from review by the Research Project Ethics Evaluation Committee of the Robert Debré Hospital (IRB 00006477).

### General Design

We performed a systematic appraisal of videos available on the internet whose main topic was the primary prevention of AHT in infants <2 years old. Because of the lack of methodological guidelines to conduct and report this type of systematic appraisal, we followed, after adaptation, the Center for Reviews and Dissemination guidance [37] and the PRISMA (Preferred Reporting Items for Systematic Review and Meta-Analysis) statement (Multimedia Appendix 1) [38].

### Search Strategy and Selection Criteria

The search was conducted on June 29, 2023. We included all videos addressing the primary prevention [39] of AHT in children <2 years old that were published in English or French (arbitrary) on the internet by public organizations (medical or scientific societies, governments or agencies, hospitals) or mainstream associations in the field of child physical abuse. We excluded commercial videos, videos not specific to children <2 years old, duplicates, and videos proposed by professionals or individuals acting outside organizations or mainstream associations.

Two investigators (a public health researcher [LG] and an educational sciences researcher [VN]) systematically searched potentially eligible videos on official websites of the national pediatric and public health societies and ministries of health of CDE [40] and on the YouTube platform, the most-used website in the world to upload and share videos [41]. On YouTube, one investigator (LG) sequentially used the terms “child physical abuse,” “shaken baby syndrome,” “abusive head trauma,” and “crying baby” in English and French to identify videos. The YouTube search engine was used in “incognito status,” with no filters, customized search methods, or personal log-in. The results were sorted by relevance according to the default YouTube search, and the first 100 videos of each search were extracted as suggested in the literature [42-45].

We first screened video titles and descriptions to identify and exclude content that was not specific to children under 2 years of age. We then evaluated each selected video in its entirety to determine its relevance to our study’s focus on AHT in infants, excluding commercial content.

### Data Extraction and Collection Process

Because of the lack of guidelines to identify the variables to be extracted in the analysis of prevention videos on the internet, the authors established a list based on previous research syntheses of health videos [45-48], public health behavior theoretical models [49-52], and AHT literature [2,3,6,10,12,15,25,53]. This list was revised and enriched after the visualization of a sample of 5 eligible videos and then discussed among researchers until a consensus was reached.

The final data extraction form included 6 sections, 28 independent items, and 3 scales (Multimedia Appendices 2-4). The first section related to the general characteristics of the videos as follows: author (public organizations or associations); author’s country (United States, France, or other); video duration; persistence (years since web publication); and the number of views, likes, and dislikes. The second section related to video creation features, including the type of narrative style used (interview, monologue, or conversational), the use of storytelling, the use of metaphor, the type of language used (colloquial and formal), and the use of audiovisual elements (sound, music, image, photograph, text or headers, subtitles, or motion design). The third section related to the characters depicted in the videos: the presence of an infant, the infant’s age (younger than 6 months), whether the infant was crying or represented being shaken, the presence of the infant’s parents and family, the professionals (legal, health, or early childhood ones) and association members involved, and the perceived origin of these characters classified as suggested by the French regulation authority for audiovisual and numeric contents [54]. The fourth section related to AHT information delivered by the videos, including the risk factors for AHT related to the infant (male sex and disability) [55] and the parents (low socioeconomic level [56], parental vulnerability [2], and poor parent-infant bonding [57]), symptoms and medical consequences of AHT (temporary or long term), characterization of shaking as an abuse, and its penal consequences. The fifth section was related to the perceived purpose of the video, which included the prevention strategies used (fear-appeal, persuasion of the severity of the depicted events, increasing the awareness of the noxious outcome of shaking, and persuasion of the effectiveness of coping strategies for infants’ cries) [49] and the perceived general outcomes searched (informational, educational, giving solution, call for action, or call to prevent from action). The sixth section included the Patient Education Materials Assessment Tool (PEMAT) Audio Visual A/V score [58,59] and the Global Quality Scale (GQS; Multimedia Appendix 4) [60].

Two investigators (LG and VN) extracted the data for each video independently. In case of disagreement between the 2 investigators, consensus was sought with the help of a third investigator (a public health researcher [EL]).

### Analyses

First, we calculated the popularity of the videos by using the Video Power Index (VPI; Multimedia Appendix 4), as suggested [61]. Second, the Cohen κ value was calculated to measure the interinvestigator agreement on each extracted variable [62]. Third, we described the characteristics of the analyzed videos. Fourth, we studied the associations between the VPI and the videos’ characteristics and GQS and PEMAT scores using first, univariate tests and correlation analyses, and then a multivariable quasi-Poisson regression (due to the overdispersion of residuals in our regression model) including all variables statistically significant (*P*<.05) in univariate analyses. The GQS and PEMAT scores were not included in the multivariable model given the redundancy of their information (Multimedia Appendix 4) with some included variables. Hypothesis tests were 2-sided, with a priori level of significance of .05. R (version 4.3.0; R Foundation for Statistical Computing) was used for all statistical analysis [63].

## Results

### Results of the Search

**Figure 1:**
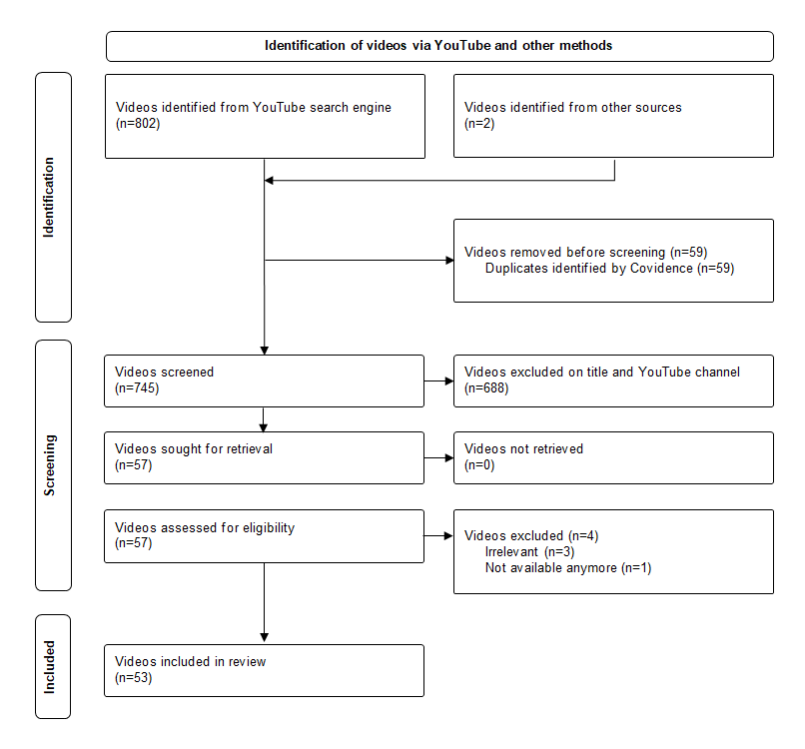
PRISMA (Preferred Reporting Items for Systematic Review and Meta-Analysis) flowchart.

Among the 804 videos identified by the search strategy, 53 (6.6%; Multimedia Appendix 5) met the inclusion criteria and were analyzed (Figure 1). All 53 videos were available on the YouTube platform; however, 2 (4%) had not been identified by our defined search on YouTube, but by searching the official websites of public organizations. The terms used to tag videos on the YouTube platform were “shaken baby syndrome” (42/53, 82%), “abusive head trauma” (11/53, 22%), and “crying baby” (2/53, 4%); none used the term “child physical abuse.”

### General Characteristics, Quality, and Popularity of Videos

Videos were published by public organizations (43/53, 81%) and associations (10/53, 19%) from the United States (34/53, 64%), France (11/53, 21%), or other countries (8/53, 15%; Table 1). They had a median length of 202 (IQR 94-333) seconds and had remained accessible via the web for a median of 6 years (IQR 3.3-9.3; Table 2). Videos accumulated a median of 7917 (IQR 1389-45,272) views, 21 (IQR 4-147) likes, and 2 (IQR 0-13) dislikes. The VPI was calculated for 48 (91%) of the 53 videos included due to missing data (number of likes and dislikes not available). The median VPI was 3 (IQR 1-14; mean 29, SD 113).

For the 28 video items rated, the median Cohen κ value was 0.65 (IQR 0.47-0.80; Multimedia Appendix 3). The videos included audiovisual elements such as text or headers (43/53, 81%), music (39/53, 74%), images (26/53, 49%), motion design (18/53, 34%), photographs (11/53, 21%), and subtitles (5/53, 9%; Table 1). Characters depicted were infants (42/53, 79%; various forms of presentation were used: real infants, photographs, motion design, and dolls), including while crying (35/53, 66%) and being shaken (21/53, 40%). The process of shaking was illustrated by motion design, the use of dolls, and metaphorical representations of an infant’s brain. Other characters included parents and family (36/53, 68%), and professionals (40/53, 75%). In 57% (30/53) of videos, these characters were from diverse origins. The cited AHT risk factors referred mainly to the infant (44/53, 83%) and then the parents (8/53, 15%). The characterization of shaking as an abuse and its penal consequences were mentioned in 47% (25/53) and 4% (2/53) of the videos, respectively. The main prevention strategies used in the videos aimed at increasing awareness of the noxious outcomes of shaking (49/53, 93%) and the persuasion of the effectiveness of coping strategies for infants’ cries (45/53, 85%).

The median understandability and actionability PEMAT scores were 83% (IQR 70%-89%) and 100% (IQR 67%-100%), respectively. The median GQS score was 4 (IQR 3-4; Table 2).

**Table 1:** Univariate associations between Video Power Index (VPI) and video characteristics.

Video characteristics						Videos (n=53), n (%)			VPI (n=48), median (IQR)			*P* value^a^
**Video creation feature**												
	**Author of the video**											.41
		Public organizations			43 (81)			3.0 (1.2-14.8)				
		Association			10 (19)			3.9 (0.7-6.9)				
	**Author’s country**											.28
		United States			34 (64)			6.6 (1.2-17.6)				
		France			11 (21)			1.9 (1.6-3.2)				
		Other			8 (15)			1.8 (0.9-5.7)				
	**Narrative style**											
		**Interview**									.96	
			Yes	20 (38)			4.8 (1.4-14.3)					
			No	33 (62)			2.9 (1.1-13.6)					
		**Monologue**									.02	
			Yes	45 (85)			3.9 (1.3-14.8)					
			No	8 (15)			0.5 (0.2-1.6)					
		**Conversational**									.41	
			Yes	14 (26)			7.3 (2.7-14.4)					
			No	39 (74)			2.3 (1.1-12.4)					
	**Use of storytelling**											.31
			Yes	12 (23)			8.0 (2.2-14.5)					
			No	41 (77)			2.7 (1.0-11.6)					
	**Use of metaphor**											.12
			Yes	4 (8)			35.1 (3.1-78.6)					
			No	49 (92)			2.9 (1.1-13.1)					
	**Type of language used**											
		**Colloquial**									.22	
			Yes	41 (77)			3.9 (1.2-14.8)					
			No	12 (19)			1.5 (0.7-7.0)					
		**Formal**									.17	
			Yes	46 (87)			4.6 (1.2-14.8)					
			No	7 (13)			1.9 (0.9-2.9)					
	**Audiovisual element**											
		**Sound**									.22	
			Yes	34 (64)			2.7 (0.9-10.5)					
			No	19 (36)			4.6 (1.8-16.5)					
		**Music**									.12	
			Yes	39 (74)			5.6 (1.3-14.4)					
			No	14 (26)			1.5 (0.6-6.1)					
		**Image**									.01	
			Yes	26 (49)			9.8 (2.8-23.7)					
			No	27 (51)			1.7 (0.7-6.6)					
		**Photograph**									.02	
			Yes	11 (21)			14.5 (8.1-23.7)					
			No	42 (79)			2.3 (0.8-8.0)					
		**Text or headers**									.02	
			Yes	43 (81)			6.5 (1.5-15.6)					
			No	10 (19)			1.1 (0.7-1.8)					
		**Subtitles**									.94	
			Yes	5 (9)			3.6 (2.4-5.5)					
			No	48 (91)			3.0 (1.1-14.8)					
		**Motion design**									.003	
			Yes	18 (34)			14.8 (3.9-25.0)					
			No	35 (66)			1.8 (0.8-6.6)					
**Characters depicted in the videos**												
	**Infant**											.07
			Yes	42 (79)			5.6 (1.3-14.8)					
			No	11 (21)			1.5 (0.7-2.9)					
	**Infant’s age younger than 6 months**											.12
			Yes	37 (70)			6.5 (1.3-14.8)					
			No	16 (30)			1.7 (1.0-3.9)					
	**Representation of an infant being shaken**											<.001
			Yes	21 (40)			13.5 (5.4-25.2)					
			No	32 (60)			1.8 (0.7-5.7)					
	**Representation of a crying infant**											.28
			Yes	35 (66)			6.5 (1.1-16.4)					
			No	18 (34)			1.8 (1.3-5.1)					
	**Infant’s parents and family**											.47
			Yes	36 (68)			6.5 (1.2-14.8)					
			No	17 (32)			2.7 (0.9-5.6)					
	**Professional**											.57
			Yes	40 (75)			3.0 (1.2-14.8)					
			No	13 (25)			3.1 (0.6-8.2)					
	**Perceived origin of the characters**											.08
		Only white			23 (43)			1.9 (0.8-6.6)				
		Mixed origin			30 (57)			6.5 (1.3-22.9)				
**AHT^b^ information delivered in the videos**												
	**Infant risk factors**											.19
			Yes	44 (83)			1.9 (1.0-13.5)					
			No	9 (17)			6.6 (2.7-16.5)					
	**Parent risk factors**											.30
			Yes	8 (15)			8.0 (3.9-17.7)					
			No	45 (85)			2.7 (1.0-12.7)					
	**Symptoms of AHT**											.049
			Yes	18 (34)			10.3 (2.6-22.3)					
			No	35 (66)			1.9 (0.9-8.1)					
	**Temporary medical consequences of AHT**											.11
			Yes	25 (47)			7.3 (1.5-16.0)					
			No	28 (53)			2.3 (0.8-7.7)					
	**Long-term medical consequences of AHT**											.17
			Yes	42 (80)			3.9 (1.2-16.0)					
			No	11 (20)			1.8 (0.8-5.8)					
	**Characterization of shaking as abuse**											.09
			Yes	25 (47)			8.0 (2.7-14.8)					
			No	28 (53)			1.8 (0.9-7.5)					
	**Penal consequences spelled out**											.86
			Yes	2 (4)			4.6 (4.6-4.6)					
			No	51 (96)			3.0 (1.1-14.5)					
**Video perceived purposes**												
	**Prevention strategy used**											
		**Fear-appeal, persuasion of severity of the depicted events**									.14	
			Yes	26 (49)			6.6 (1.2-18.8)					
			No	27 (51)			1.9 (1.1-8.0)					
		**Increasing the awareness of the noxious outcome of shaking**									.06	
			Yes	49 (93)			3.9 (1.2-14.8)					
			No	4 (7)			0.9 (0.0-2.1)					
		**Persuasion of the effectiveness of coping strategies for infants’ cries**									.68	
			Yes	45 (85)			3.0 (0.9-14.8)					
			No	8 (15)			3.7 (2.3-7.9)					
	**Perceived general outcome searched**											
		**Educational**									.91	
			Yes	49 (93)			3.1 (1.1-14.8)					
			No	4 (7)			2.7 (2.4-4.1)					
		**Informational**									.37	
			Yes	50 (94)			3.1 (1.2-14.2)					
			No	3 (6)			0.2 (0.1-56.6)					
		**Giving** **solution**									.56	
			Yes	43 (81)			3.1 (1.1-14.8)					
			No	10 (19)			2.7 (1.2-6.1)					
		**Call for action**									.12	
			Yes	43 (81)			4.9 (1.2-16.0)					
			No	10 (19)			2.0 (0.9-4.1)					
		**Call to prevent from action**									.38	
			Yes	48 (91)			3.0 (1.2-14.8)					
			No	5 (9)			3.1 (0.7-6.6)					

^a^Mann-Whitney and Kruskal-Wallis tests.

^b^AHT: abusive head trauma.

**Table 2:** General characteristics of the included videos and association with the Video Power Index (VPI).

Video features	Value (n=53), n (%)	Value (n=48), median (IQR)	*r* (n=48)	*P* value^a^ (n=48)
Duration (seconds)	53 (100)	202 (94-333)	0.36	.01
Number of years accessible via the web	53 (100)	6 (3-9)	N/A^b^	N/A
Number of views	53 (100)	7917 (1389-45,272)	N/A	N/A
Number of likes	48 (91)	21 (4-147)	N/A	N/A
Number of dislikes	48 (91)	2 (0-13)	N/A	N/A
VPI	48 (91)	3 (1-14)	N/A	N/A
GQS^c^ score	53 (100)	4 (3-4)	0.38	.007
PEMAT^d^ understandability score (%)	53 (100)	83 (70-89)	0.27	.06
PEMAT actionability score (%)	53 (100)	100 (67-100)	0.27	.07

^a^Spearman correlation test.

^b^Not applicable.

^c^GQS: Global Quality Scale.

^d^PEMAT: Patient Education Materials Assessment Tool.

### Determinants of Popularity

In the univariate analyses (Table 1), only monologue used as narrative style was significantly associated with increased VPI (median 3.9 vs 0.5 for no monologue; *P*=.02). The VPI was significantly increased when videos contained images (median 9.8 vs 1.7; *P*=.01), photographs (median 14.5 vs 2.3; *P*=.02), text or headers (median 6.5 vs 1.1; *P*=.02), and motion design (median 14.8 vs 1.8; *P*=.003) as audiovisual elements. The presence of an infant in the videos was not associated with the VPI. The VPI was significantly increased with the depiction of an infant being shaken (median 13.5 vs 1.8; *P*<.001). Character types represented in the video (infant’s parents and family, professionals) and their perceived origin were not significantly associated with the VPI (all *P*≥.05). The VPI was significantly increased when symptoms of AHT were mentioned (median 10.3 vs 1.9; *P*=.049) but not when shaking was characterized as an abuse (*P*=.09) or when its penal consequences were stated (*P*=.86). None of the prevention strategies used in the videos and the perceived video’s general outcomes were associated with increased VPI. The VPI was significantly positively correlated with the duration of the videos (*r*=0.36; *P*=.01) and the GQS score (*r*=0.38; *P*=.007) but not PEMAT scores (all *P*≥.05).

In the multivariable analysis (Table 3), increased VPI was independently significantly associated only with the use of text or headers in the videos (*P*=.04; β=2.15, 95% CI 1.08-4.26) and the representation of an infant being shaken (*P*=.03; β=1.74, 95% CI 1.06-2.85).

**Table 3:** Multivariable analysis of the association of variables with Video Power Index (n=48).

Video features			β		95% CI		*P* value
**Duration (seconds)**			1.00		1.00-1.00		.60
**Narrative style: monologue**			2.44		0.98-6.10		.06
**Audiovisual element**							
	Image	0.85		0.50-1.45		.55	
	Photograph	1.26		0.79-2.02		.34	
	Text or headers	2.15		1.08-4.26		.04	
	Motion design	1.00		0.58-1.75		.99	
**Representation of an infant being shaken**			1.74		1.06-2.85		.03
**Symptoms of abusive head trauma**			1.35		0.85-2.13		.21

## Discussion

### Principal Findings

This systematic appraisal highlights the relationship between the characteristics and content of health web-based prevention videos and their popularity. It leads to practical implications for developing more effective prevention campaigns. In our study, VPIs were significantly increased for videos that included images, photographs, text or headers, and motion design. Although numerous studies examined the popularity of preventive health videos or health topics, none analyzed the relation between visual elements within the videos and their popularity [46,47,64-66]. In another educational field, instructional videos, one study had the same conclusion as ours on the determinants of popularity [67]. In our study, the representation of an infant being shaken was associated with increased VPI, a result providing additional evidence to support considering the use of shocking content on this topic. Shocking content is often used in prevention campaigns because it is supposed to better capture the audience’s attention [68-70], but there is no consensus on its effectiveness [68-72]. Finally, in our study, as in other studies in various fields [73-75], the quality of the videos was positively correlated with their popularity.

In our study, the overall content of 53 videos of AHT prevention had good quality as indicated by a median GQS score of 4. This result can be explained in part by the videos being mainly published by public organizations, whose videos generally have higher GQS score than those published by nonprofessionals [48,64,76]. The videos we analyzed mostly contained information about the symptoms and medical consequences of AHT as well as the dangerous consequences of shaking an infant. This observation is consistent with the main content of AHT prevention interventions that do not use the internet [16,21]. The videos were mostly educational and used a strategy of persuasion about the effectiveness of coping, which is consistent with the strategies used in most AHT prevention interventions that attempt to convince parents of the efficacy of coping strategies in managing the response to infant crying (eg, walk away, leave the infant alone, or call a support person) [16,21]. However, some information was not addressed, particularly regarding parental risk factors, a finding similar to the content of other AHT prevention interventions [16,21]. By focusing solely on child risk factors and neglecting parental and more generally perpetrator risk factors, the videos analyzed could induce a deleterious shift of the responsibility for shaking from the adults to the infant, a mechanism similar to victim blaming [77-79]. Including perpetrators’ risk factors in prevention programs would help raise awareness of the personal responsibility of perpetrators and limit their ability to claim to be victims themselves, passive and will-less in their wrongdoing [78]. The characterization of shaking as an abuse and its penal consequences were often missing in the analyzed videos and were not found to be associated with popularity. The use of these arguments in prevention campaigns is debated. Emphasizing legal consequences might decrease audience participation in prevention campaigns, particularly because of stigma [80,81]. However, some studies suggest that education about the legality, consequences, and responsibilities associated with abusive behavior might enhance the target audience’s understanding and commitment to preventing such behavior [82-84]. The optimal balance between legal deterrence and educational strategies should be adapted according to the objectives of the prevention campaigns.

The analyzed videos had a mean VPI of 29. There is no validated threshold to interpret a VPI value. The mean VPI in our study seems to be rather low compared with VPIs reported in the medical literature, which range from <1 (eg, 0.17 in health prevention videos) [46] to >1000 (eg, 1078 in chronic medical conditions videos) [47,64-66], and are lower when the publisher is a public organization [46,64,66]. The video platform that we used, YouTube, may itself use a recommendation algorithm that will not prioritize or suggest preventive AHT content because of potential violent content linked to the representation of a shaken infant. Furthermore, only 4% (2/53) of the analyzed videos resulted from the search term “crying baby,” whereas crying is reported as the primary trigger in most perpetrations of AHT [11] and is probably the most frequent term used by parents rather than “shaken baby syndrome,” the most frequent tag used in the analyzed videos. In the future, publishers should systematically tag their videos with the term “crying baby”.

### Limitations

The first limitation is that we included only videos published by public organizations in CDE and mainstream associations, on YouTube and on public organization websites, in English and French. As well, the search was restricted to the first 100 videos for each search term. The characteristics and popularity of videos posted on other popular platforms, such as Facebook, Instagram, and TikTok, and by other publishers may differ. Our findings, which are limited to videos using English and French languages, may not be generalized to all contexts. The direction and magnitude of the probable selection biases introduced by these criteria are unknown. Second, because of these strict selection criteria, the sample size resulted in limited statistical power to identify the determinants of VPI. Third, the median Cohen κ indicated only substantial agreement between investigators, thus highlighting the subjectivity of quoted criteria. Finally, although the GQS and VPI are increasingly used tools in the literature, they are not validated and there is no threshold for their interpretation.

### Implications

As a first step to developing an effective AHT prevention program, this systematic appraisal of available videos suggests that developing high-quality content for AHT prevention videos can have a significant positive impact on their popularity. Future videos may also include specific features such as text or headers and the depiction of an infant being shaken to improve their popularity. However, popularity alone does not ensure the effectiveness of these videos in preventing AHT. Therefore, understanding popularity must be complemented by evaluating the effectiveness of the content and the needs and expectations of the target audience. Increasing popularity could lead to more impactful prevention programs.

Another implication is the need for the development and validation of methodological tools to scientifically conduct and report health video synthesis research. Videos will probably become the main channel for public health programs in the coming years, and the standardization of methods is critical. Currently, there is an urgent need to validate or refine the PEMAT, GQS, and VPI as tools to evaluate the quality and popularity of videos. Furthermore, consensus is needed on core items that must be evaluated when analyzing videos. Methods to maximize the objectivity of the assessments should also be produced and made available to the research community.

By establishing an association between video quality and popularity, this research provides insights for the development of more effective digital prevention strategies that could be of use beyond AHT. The methodology developed to study the videos, in the absence of a recommended approach, could serve as a basis for further methodological developments that would improve research practices in this field.

### Conclusion

Videos addressing the primary prevention of AHT in children <2 years old that were published on the internet in English or French by public organizations or mainstream associations were of high quality but did not frequently deal with parental risk factors. The inclusion of characteristics identified as associated with the popularity of AHT prevention videos could help improve the impact of future prevention programs by enhancing their popularity. Finally, there is an urgent need for the development and validation of methodological tools to standardize the conduct and report of studies on prevention videos.

## Appendix

Multimedia Appendix 1 PRISMA (Preferred Reporting Items for Systematic Review and Meta-Analysis) checklist.

Multimedia Appendix 2 Metrics of the videos analyzed.

Multimedia Appendix 3 Video characteristics and their κ coefficient for investigator agreement.

Multimedia Appendix 4 Description of the Global Quality Scale (GQS), Patient Education Materials Assessment Tool Audio Visual (PEMAT) A/V scale, and Video Power Index (VPI) .

Multimedia Appendix 5 Videos included in the appraisal.

Multimedia Appendix 6 Data sharing statement.

## Abbreviations

AHT: abusive head trauma
CDE: countries with developed economies
GQS: Global Quality Scale
PEMAT: Patient Education Materials Assessment Tool
PRISMA: Preferred Reporting Items for Systematic Review and Meta-Analysis
VPI: Video Power Index

## Data Availability

The datasets generated and analyzed during this study are available from the corresponding author on reasonable request (Multimedia Appendix 6).
