# Supplementary material for: Characteristics and Popularity of Videos of Abusive Head Trauma Prevention: Systematic Appraisal
Source: J Med Internet Res. 2024 Dec 10;26:e60530. doi: 10.2196/60530 (PMC11668989; doi:10.2196/60530)
Supplement: Multimedia Appendix 2 [file jmir_v26i1e60530_app2.docx]

**Multimedia Appendix 2: Metrics of the videos analyzed**

| **Variable** | **Definition** |
| --- | --- |
| Duration (seconds) | The length of the video in seconds. |
| Number of years online | The duration of time that the video has been available online. |
| Number of views | The total count of views the video has received. |
| Number of likes | The total count of positive reactions or likes for the video. |
| Number of dislikes | The total count of negative reactions or dislikes for the video. |
