# Supplementary material for: Characteristics and Popularity of Videos of Abusive Head Trauma Prevention: Systematic Appraisal
Source: J Med Internet Res. 2024 Dec 10;26:e60530. doi: 10.2196/60530 (PMC11668989; doi:10.2196/60530)
Supplement: Multimedia Appendix 3 [file jmir_v26i1e60530_app3.docx]

**Multimedia Appendix 3: Video characteristics and their kappa coefficient for investigator agreement**

| **Variable** | **Definition** | **K** |
| --- | --- | --- |
| ***Video creation feature*** | |  |
| Author of the video | The individual or entity responsible for creating and producing the video content: public organizations (medical or scientific societies, governments or agencies, hospitals) or mainstream associations in the field of child physical abuse | NC |
| Author’s country | United States, France, and other | NC |
| Narrative style |  |  |
| -- Interview | A narrative approach characterized by conveying information through a structured conversation or dialogue, typically involving questions and answers, to provide a comprehensive understanding of a subject or share personal insights. | 0.86 |
| -- Monologue | A narrative approach characterized by an expressive and self-contained speech or presentation where by an individual shares a narrative or story, often delivering it in a continuous and uninterrupted manner. | 0.93 |
| -- Conversational | A narrative approach characterized by an informal and interactive dialogue, resembling a conversation, to present information or tell a story in a more engaging and approachable manner. | 0.89 |
| Use of storytelling | A method of communication that involves recounting a series of events or experiences in a cohesive and engaging manner to convey a message or evoke emotions | 0.94 |
| Use of metaphor | Using metaphorical language and symbolic comparisons within a narrative to convey abstract ideas or complex concepts in a more vivid and relatable manner. | 0.64 |
| Type of language used | |  |
| -- Colloquial | Informal language characterized by everyday speech, commonly used in casual conversations. | 0.59 |
| -- Formal | Polished and structured language suitable for official or professional communication. | 0.64 |
| Audiovisual element |  |  |
| -- Sound | Supplementary audio components added to a video to enhance its auditory experience, encompassing elements such as ambient sounds, or special effects. | 0.26 |
| -- Music | Additional musical elements integrated into a video to enrich the emotional atmosphere, establish a specific mood, or complement the visual content. | 0.74 |
| **Variable** | **Definition** | **K** |
| ***Audiovisual element (continued)*** | |  |
| -- Image | Extra visual representations or graphic elements inserted into a video to convey information, add visual interest, or evoke particular sentiments. | 0.53 |
| -- Photograph | Added still images captured by a camera, utilized in a video to visually communicate information or enhance storytelling. | 0.59 |
| -- Text or headers | Inserted written content or titles within a video to provide context, deliver information, or introduce different sections of the content. | 0.75 |
| -- Subtitles | Textual additions displayed on screen during a video, serving purposes such as translating spoken dialogue or improving accessibility for individuals with hearing impairments. | 0.10 |
| -- Motion design | Additional dynamic visual elements like animations and graphics incorporated into a video to elevate its visual appeal and engagement. | 0.85 |
| ***Characters depicted in the videos*** | |  |
| Infant | Presence or representation of an infant character in the video. | 0.40 |
| Infant’s age under 6 months | The age category indicating whether the infant character in the video is under 6 months old. | 0.56 |
| Representation of an infant being shaken | Depiction or representation of an infant being shaken in the video. | 0.83 |
| Representation of a crying infant | Depiction or representation of a crying infant in the video. | 0.80 |
| Infant’s parents and family | Presence or representation of parental and family figures in the video. | 1.0 |
| Professional | Presence or representation of a professional character in the video. |  |
|  | Health professional | 0.31 |
|  | Early childhood professional | 0.44 |
|  | Member of an association | 0.65 |
|  | Legal professional | 0.49 |
| Perceived origin of the characters | The origin of the characters, classified as "Only white" or "Mixed origin." |  |
|  | White | 0.47 |
|  | Black | 0.88 |
|  | Arab | 0.17 |
|  | Asian | 0.75 |
|  | Not identified | 0.25 |
|  | Other | 0.12 |
| ***AHT information delivered in the videos*** | |  |
| Infant’s risk factor | The presence or mention of risk factors affecting the infant in the video. |  |
|  | Inconsolable crying | 0.55 |
|  | Sleep disorder | 0.40 |
|  | Feeding difficulties | 0 |
|  | Male sex | 1 |
| Parent’s risk factor | The presence or mention of risk factors associated with the parents in the video. |  |
|  | Young maternal age | 0.66 |
|  | Late prenatal care start | NC |
|  | Psychological distress | 0.20 |
|  | Economic deprivation | 0.79 |
|  | Psychiatric disorder | NC |
|  | Intimate partner violence | 0.66 |
|  | Prematurity | NC |
|  | Multiple pregnancy | NC |
|  | Unwanted pregnancy | NC |
| Symptoms of AHT | Indication or mention of symptoms related to AHT affecting the infant. | 0.71 |
| Temporary medical consequences of AHT | Mention of temporary medical consequences or after-effects for the infant resulting from specific actions in the video. | 0.83 |
| Long-term medical consequences of AHT | Mention of long-term medical consequences for the infant resulting from specific actions in the video. | 0.73 |
| Characterization of shaking as an abuse | Recognition or portrayal of shaking as an abusive action towards the infant in the video. | 0.64 |
| Penal consequences spelled out | Explicit mention or explanation of the legal framework and potential legal consequences associated with actions in the video. | 1.00 |
| ***Video perceived purposes*** | |  |
| Prevention strategy used |  |  |
| -- Fear–appeal persuasion of severity of the depicted events | Inducing the emotion of fear to better persuade individuals by emphasizing the severity of potential events depicted, aiming to encourage preventive behaviors. | 0.75 |
| -- Increasing the awareness of the noxious outcome of shaking | Strategies aimed at raising awareness regarding the harmful outcomes associated with a particular situation, intending to promote preventive actions. | 0.47 |
| -- Persuasion of the effectiveness of coping strategies | Attempt to convince individuals about the efficacy of coping strategies in managing or mitigating the negative consequences of a given situation, with the goal to promote proactive preventive measures. | 0.64 |
| General outcome |  |  |
| -- Educational | The educational impact or result intended by the video, emphasizing the imparting of knowledge or information. | NC |
| -- Informational | The informational impact or result intended by the video, providing facts or details to the audience. | 0.73 |
| -- Giving solution | The presentation of solutions or resolutions to address a particular issue or problem in the video. | 0.93 |
| -- Call for action | The intended impact is to motivate viewers to actively participate in a specific behavior, initiative, or movement suggested in the video. | 0.65 |
| -- Call to prevent from action | The intended impact is to discourage viewers from engaging in certain actions by raising awareness about potential negative consequences and encouraging preventive measures. | 0.56 |

 NC, not calculable
