# Supplementary material for: Characteristics and Popularity of Videos of Abusive Head Trauma Prevention: Systematic Appraisal
Source: J Med Internet Res. 2024 Dec 10;26:e60530. doi: 10.2196/60530 (PMC11668989; doi:10.2196/60530)
Supplement: Multimedia Appendix 4 [file jmir_v26i1e60530_app4.docx]

**Multimedia Appendix 4: Description of Global Quality Scale (GQS), Patient Education Materials Assessment Tool Audio Visual (PEMAT) A/V scale and Video Power Index (VPI)**

| **Tool** | **Score** | **Variables used to assess** | **Interpretation** |
| --- | --- | --- | --- |
| The GQS, with a score ranging from 1 to 5, was used to measure the usefulness and quality of the video [60]. We interpreted the GQS as suggested: 1 or 2 points indicate low quality, 3 medium quality, and 4 or 5 high quality. | 1-5 | Representation of an infant being shaken  Representation of a crying infant  infant’s risk factor  Parent’s risk factor  Symptoms of AHT  Temporary medical consequences of AHT  Long-term medical consequences of AHT  Characterization of shaking as an abuse  Penal consequences spelled out | Higher values indicate higher quality |
| The PEMAT is a validated 20-item tool developed by the Agency for Healthcare Research and Quality [58, 59] for measuring understandability and actionability. Each item is awarded 1 for "agree" and 0 for "disagree or not applicable,” then scores are aggregated and converted to a percentage. A threshold of 70% is proposed as an indicator of understandable and actionable information [58, 59] | 0-100% |  | Higher percentage indicates higher understandability and actionability |
| The VPI assesses the view ratio (number of views/number of days since publication) and the like ratio (like*100/[like+dislike)) of each video using the following formula: like ratio×view ratio/100 [61]. A higher VPI means greater popularity. No threshold is proposed to interpret the VPI. To collect data on dislikes, we use the Return YouTube Dislike Chrome extension because of YouTube’s policy of concealing dislikes, which went into effect in November 2021. |  | Formula: [(view ratio × like ratio/100), where view ratio = views/day and like ratio = [(likes × 100)/(likes + dislikes)] | A higher VPI means greater popularity |
