# Supplementary material for: Characteristics and Popularity of Videos of Abusive Head Trauma Prevention: Systematic Appraisal
Source: J Med Internet Res. 2024 Dec 10;26:e60530. doi: 10.2196/60530 (PMC11668989; doi:10.2196/60530)
Supplement: Multimedia Appendix 5 [file jmir_v26i1e60530_app5.docx]

**Multimedia Appendix 5: Videos included in the appraisal**

| **YouTube channel** | **Title** | **YouTube link** | **GQS** | **VPI** |
| --- | --- | --- | --- | --- |
| Bundeszentrale für gesundheitliche Aufklärung (BZgA) | Quand les bébés crient : comment les consoler et les calmer | https://www.youtube.com/watch?v=NHCFodVt20s&t=1s | 3 | 0.0 |
| PowertoProtect | Power to Protect. Never, ever shake an infant | https://www.youtube.com/watch?v=bM2yc3oRu8g | 4 | NA |
| ProjectChildSafetyVideos | Shaken Baby Syndrome (Abusive Head Trauma) | https://www.youtube.com/watch?v=q6xNjaK2F9w | 3 | 0.7 |
| PCAKY | Safe Sleep Practices & Pediatric Abusive Head Trauma Prevention | https://www.youtube.com/watch?v=VixgUYwQiIY | 5 | 6.5 |
| FrederickCountyMD | Focus on Health: Shaken Baby Syndrome (Abusive Head Trauma) | https://www.youtube.com/watch?v=dyTYTapuCG8 | 4 | 0.2 |
| NortonChildren's | Pediatric Abusive Head Trauma Training \| Prevention & Wellness | https://www.youtube.com/watch?v=EyHOogy-oH4 | 3 | 0.0 |
| SaintAlsHealth | Preventing Child Head Trauma: Crying Connection | https://www.youtube.com/watch?v=CNc4SocWkak | 5 | 8.0 |
| MissouriCTF | Countdown to Calm: Preventing Abusive Head Trauma | https://www.youtube.com/watch?v=OVnlqsTBsAg | 4 | 6.5 |
| RoperSt | Preventing Abusive Head Trauma: The Dangers of Shaking an Infant | https://www.youtube.com/watch?v=okbFBEZrmws | 4 | 24.5 |
| NortonChildren's | Pediatric Abusive Head Trauma Training 2 \| Prevention & Wellness | https://www.youtube.com/watch?v=7sk5_zEOmTQ | 3 | 0.2 |
| countysandiego | Preventing Abusive Head Trauma | https://www.youtube.com/watch?v=F1UwcmtvVfo | 4 | 1.4 |
| **YouTube channel** | **Title** | **YouTube link** | **GQS** | **VPI** |
| NCT | How to Cope with a Crying Baby | https://www.youtube.com/watch?v=nqZ7TmE2B9g | 4 | 8.4 |
| UCDavisHealth | How to Soothe a Crying Baby - Tips for New Parents and Caregivers | https://www.youtube.com/watch?v=aP33Xn6T3Vc | 4 | 41.6 |
| ChildCareLicensingUtah | CCL - Prevention of Shaken Baby Syndrome | https://www.youtube.com/watch?v=v4BRCG5jL6M | 4 | NA |
| LucasCountyProsecutor | Shaken baby syndrome prevention PSA | https://www.youtube.com/watch?v=IwOM6NMl-Xw | 1 | 0.8 |
| StopShakenBabySyndrome | Keep Your Cool - PSA for Preventing Shaken Baby Syndrome | https://www.youtube.com/watch?v=ald5pBFgcLs | 2 | 0.4 |
| AFMSCEMMTube | Preventing Shaken Baby Syndrome | https://www.youtube.com/watch?v=VkzFsnjFRPY | 3 | 1.3 |
| ClevelandClinic | Newborn Care and NICU Baby Guide for Parents \| Shaken Baby Syndrome Prevention | https://www.youtube.com/watch?v=6JrfCyXeS2M | 4 | 1.9 |
| StopShakenBabySyndrome | Stop Shaken Baby Syndrome - Campaign Video | https://www.youtube.com/watch?v=53MHcAXnbf8 | 3 | 1.2 |
| Scott&WhiteHealthcare | Preventing Shaken Baby Syndrome | https://www.youtube.com/watch?v=0w5dfqg7Dxc | 4 | 1.1 |
| westchesterDA | Shaken Baby Syndrome | https://www.youtube.com/watch?v=m9m5KwvX0pQ | 3 | 12.7 |
| AFMSCEMMTube | Shaken Baby Syndrome Facts | https://www.youtube.com/watch?v=DaS5L2KKtvA | 2 | 2.7 |
| scdhec | Understanding Shaken Baby Syndrome | https://www.youtube.com/watch?v=j8sNRsnAOZM | 4 | 3.0 |
| **YouTube channel** | **Title** | **YouTube link** | **GQS** | **VPI** |
| NYSDOH NY | Never, Ever, Shake a Baby: A Video for Day Care Providers | https://www.youtube.com/watch?v=Njx5NdNSfsY | 4 | 21.2 |
| BrainInjuryAssnUtah | TBI in Young Children 4: Shaken Baby Syndrome and Normal Infant Crying | https://www.youtube.com/watch?v=6tEm2u-Epr0 | 4 | 1.7 |
| NYSDOH | Never, Ever, Shake a Baby: A Video for New Parents | https://www.youtube.com/watch?v=CjyhfIZKmSg | 4 | 25.5 |
| AFMSCEMMTube | Immediate Shaken Baby Syndrome Symptoms | https://www.youtube.com/watch?v=Y9zQO8rjbv4 | 4 | 6.6 |
| MissouriCTF | Never Shake: Preventing Shaken Baby Syndrome (English) | https://www.youtube.com/watch?v=M3xytsg6oDo | 5 | 67.1 |
| BoysTownHospital | Shaken Baby Syndrome - Boys Town Pediatrics | https://www.youtube.com/watch?v=I8v_Ip30XEw | 4 | 16.5 |
| Madisonhealth | Shaken Baby Syndrome | https://www.youtube.com/watch?v=PYJZ2ib3aTQ | 2 | 11.6 |
| NYSDOH | Never, Ever, Shake a Baby: A Video for New Dads | https://www.youtube.com/watch?v=5MA1HqsPJOo | 5 | 14.8 |
| PhoenixChildren’s | Never Shake a Baby - Phoenix Children's Hospital | https://www.youtube.com/watch?v=hM_BE4mz0ss | 4 | 113.1 |
| Nebraska Methodist Health System | Shaken Babies: Shattered Dreams | https://www.youtube.com/watch?v=Ju83qKTvDzw | 4 | 14.2 |
| NebraskaDHHS | Shaken Baby Syndrome | https://www.youtube.com/watch?v=VLBu_OkBmfQ | 4 | NA |
| AFMSCEMMTube | Shaken Baby Syndrome Brain Injury | https://www.youtube.com/watch?v=YoXonRCHM1w | 3 | 98.9 |
| **YouTube channel** | **Title** | **YouTube link** | **GQS** | **VPI** |
| LomaLindaUniversityHealth | It is never ok to shake a baby | https://www.youtube.com/watch?v=ddVwDXht7AU | 5 | 780.0 |
| SoutheastGeorgiaHealthSystem | Shaken Baby Syndrome | https://www.youtube.com/watch?v=F393zg8Q-r0 | 3 | 14.8 |
| Hôpital Armand Trousseau AP-HP | Prévention du syndrome du bébé secoué | https://www.youtube.com/watch?v=4lqK0W998Jo | 4 | 3.2 |
| BertrandGIMONET | Spot de prévention du syndrome du bébé secoué | https://www.youtube.com/watch?v=tWMWwL2rqVk | 3 | 2.7 |
| Bundeszentrale für gesundheitliche Aufklärung (BZgA) | Ne jamais secouer ! Lorsque les bébés ne cessent pas de crier | https://www.youtube.com/watch?v=YOsNyn6KmAs | 5 | 1.00 |
| HAS - Haute Autorité de santé | HAS - Bébé secoué : Les réponses du médecin | https://www.youtube.com/watch?v=IscZPl8f4oU | 3 | 1.6 |
| HAS - Haute Autorité de santé | HAS–Bébé secoué : Ce que savent les jeunes parents | https://www.youtube.com/watch?v=-9O4Go_lidQ | 3 | 1.9 |
| Stopbébésecoué | Confinement : Attention au Syndrome du bébé secoué | https://www.youtube.com/watch?v=qNPQSSSkFwM | 3 | NA |
| syndromedubebesecoue | Qu'est ce que le syndrome du bébé secoué ? | https://www.youtube.com/watch?v=U3thbSjOxxE | 2 | 0.7 |
| VilledeNice-Nice | Vidéo de prévention contre le syndrome du bébé secoué - 2023 | https://www.youtube.com/watch?v=Zd8sIwPHa3c | 3 | 4.6 |
| HopitauxRobertSchuman | Syndrome du bébé secoué : comment rassurer son bébé | https://www.youtube.com/watch?v=z8Ucq_mbfg0 | 4 | 0.8 |
| syndromedubebesecoue | Le Syndrome du bébé secoué combattre les idées reçues | https://www.youtube.com/watch?v=7W_BpaIR6yU | 3 | NA |
| **YouTube channel** | **Title** | **YouTube link** | **GQS** | **VPI** |
| HopitauxRobertSchuman | Syndrome du bébé secoué: angoisse face aux pleurs de bébé | https://www.youtube.com/watch?v=Rt-h-kttG-Q | 4 | 1.8 |
| HopitauxRobertSchuman | Syndrome du bébé secoué : comment détendre et apaiser bébé | https://www.youtube.com/watch?v=mfHuUM41XBo | 4 | 3.1 |
| Lenval | Prévention face au syndrome du bébé secoué - Dr Chivoret - Fondation Lenval | https://www.youtube.com/watch?v=-2nRsTjGZ5A | 3 | 1.7 |
| HopitauxRobertSchuman | Syndrome du bébé secoué - De quoi s'agit-il ? | https://www.youtube.com/watch?v=wEPiadyBmzY | 4 | 49.9 |
| Tatiana Soutien Prévention Syndrome Bébé Secoué | Spot de prévention Syndrome du bébé secoué "Secouer n'est pas calmer" | https://www.youtube.com/watch?v=-UYovw1_dRA | 3 | 8.0 |
| lesmauxlesmotspourledire | Prévention du syndrome du bébé secoué ou SBS | https://www.youtube.com/watch?v=qUiKKd2gFFg | 4 | 0.6 |
