# Supplementary material for: Characteristics and Popularity of Videos of Abusive Head Trauma Prevention: Systematic Appraisal
Source: J Med Internet Res. 2024 Dec 10;26:e60530. doi: 10.2196/60530 (PMC11668989; doi:10.2196/60530)
Supplement: Multimedia Appendix 6 [file jmir_v26i1e60530_app6.docx]

**Multimedia Appendix 6**

**DATA SHARING STATEMENT**

**Data**

Data available: Yes

Data types: YouTube video’s characteristics

How to access data: All data used for this systematic appraisal are on the YouTube platform, but a copy of the specific datasets used can be accessed by contacting the corresponding author.

When available: With publication

**Supporting documents**

Document types: None

**Additional information**

Who can access the data: Anyone requesting the data

Types of analyses: For any purpose

Mechanisms of data availability: With investigator support
